# Expert recommendations for implementing change (ERIC): protocol for a mixed methods study

**DOI:** 10.1186/1748-5908-9-39

**Published:** 2014-03-26

**Authors:** Thomas J Waltz, Byron J Powell, Matthew J Chinman, Jeffrey L Smith, Monica M Matthieu, Enola K Proctor, Laura J Damschroder, JoAnn E Kirchner

**Affiliations:** 1 Department of Veterans Affairs Medical Center, 2200 Fort Roots Drive (152/NLR), Central Arkansas Veterans Healthcare System, HSR&D and Mental Health Quality Enhancement Research Initiative (QUERI), Little Rock, Arkansas, USA; 2 Department of Psychology, 301D Science Complex, Eastern Michigan University, Ypsilanti, MI, USA 48197; 3 Brown School, Washington University in St. Louis, St. Louis, Missouri, USA; 4 Veterans Research and Education Foundation of Saint Louis, d.b.a. Vandeventer Place Research Foundation, St. Louis, Missouri, USA; 5 VISN 4 MIRECC, Pittsburgh, Pennsylvania, USA; 6 RAND Corporation, Pittsburgh, Pennsylvania, USA; 7 School of Social Work, College for Public Health & Social Justice, Saint Louis University, St. Louis, Missouri and St. Louis VA Health Care System, St. Louis, USA; 8 HSR&D Center for Clinical Management Research, VA Ann Arbor Healthcare System, Ann Arbor, Michigan, USA; 9 Department of Psychiatry, College of Medicine, University of Arkansas for Medical Sciences, Little Rock, Arkansas, USA

**Keywords:** Implementation research, Implementation strategies, Mixed methods, U.S. Department of Veterans Affairs

## Abstract

**Background:**

Identifying feasible and effective implementation strategies that are contextually appropriate is a challenge for researchers and implementers, exacerbated by the lack of conceptual clarity surrounding terms and definitions for implementation strategies, as well as a literature that provides imperfect guidance regarding how one might select strategies for a given healthcare quality improvement effort. In this study, we will engage an Expert Panel comprising implementation scientists and mental health clinical managers to: establish consensus on a common nomenclature for implementation strategy terms, definitions and categories; and develop recommendations to enhance the match between implementation strategies selected to facilitate the use of evidence-based programs and the context of certain service settings, in this case the U.S. Department of Veterans Affairs (VA) mental health services.

**Methods/Design:**

This study will use purposive sampling to recruit an Expert Panel comprising implementation science experts and VA mental health clinical managers. A novel, four-stage sequential mixed methods design will be employed. During Stage 1, the Expert Panel will participate in a modified Delphi process in which a published taxonomy of implementation strategies will be used to establish consensus on terms and definitions for implementation strategies. In Stage 2, the panelists will complete a concept mapping task, which will yield conceptually distinct categories of implementation strategies as well as ratings of the feasibility and effectiveness of each strategy. Utilizing the common nomenclature developed in Stages 1 and 2, panelists will complete an innovative menu-based choice task in Stage 3 that involves matching implementation strategies to hypothetical implementation scenarios with varying contexts. This allows for quantitative characterizations of the relative necessity of each implementation strategy for a given scenario. In Stage 4, a live web-based facilitated expert recommendation process will be employed to establish expert recommendations about which implementations strategies are essential for each phase of implementation in each scenario.

**Discussion:**

Using a novel method of selecting implementation strategies for use within specific contexts, this study contributes to our understanding of implementation science and practice by sharpening conceptual distinctions among a comprehensive collection of implementation strategies.

## Background

Implementation research is a promising means of improving the quality of mental healthcare delivery, both by increasing our understanding of determinants of practice (*i.e.*, barriers and facilitators) that can influence organizational, provider and patient behavior, and by building an evidence base for specific implementation strategies that can move evidence-based programs and practices (EBPPs) into routine care [1,2]. It has particular utility within contexts such as the U.S. Department of Veterans Affairs (VA), in which the use of EBPPs has been mandated via requirements set forth in the Uniform Mental Health Services Handbook [3]. The VA’s Quality Enhancement Research Initiative (QUERI) has outlined a number of steps for advancing implementation research within VA [4]. These steps include: selecting conditions associated with a high risk of disease, disability, and/or burden of illness; identifying evidence-based guidelines, recommendations, and best practices; measuring and diagnosing quality and performance gaps; implementing improvement programs; and evaluating improvement programs [4]. The fourth step in this process, implementing improvement programs, requires identifying, developing, or adapting implementation strategies and deploying them to improve the quality of care delivery [4]. Yet, identifying implementation strategies that are feasible and effective to get a given practice change into wide use in clinical settings with varying contexts remains a challenge for researchers and implementers within VA and beyond. The Expert Recommendations for Implementing Change (ERIC) process was developed to address two major limitations of the published literature: lack of conceptual clarity with regard to implementation strategies and insufficient guidance about how to select appropriate strategies for implementing a particular EBPP in a particular context.

### Lack of conceptual clarity for implementation strategies

The lack of clarity in terminology and definitions in the implementation literature has been well-documented [5-8]. Frequently, terms and definitions for implementation strategies are inconsistently applied [5,9], and they are rarely defined or described in sufficient detail to be useful to implementation stakeholders [6,10]. The inconsistent use of terms and definitions can involve homonymy (*i.e.*, same term has multiple meanings), synonymy (*i.e.*, different terms have the same, or overlapping meanings), and instability (*i.e.*, these terms shift unpredictably over time) [10,11]. For example, Kauth *et al.*[12] note that ‘terms such as *educator*, *academic detailer*, *coach*, *mentor*, *opinion leader*, and *champion* are often confused with *facilitator*’, (italics in original) and are not differentiated from each other despite important conceptual distinctions. The inconsistency of implementation strategy terms and definitions complicates the acquisition and interpretation of research literature, precludes research synthesis (*e.g.*, systematic reviews and meta-analyses), and limits capacity for scientific replication [6,13]. The challenges associated with the inconsistent labeling of terms is compounded by the fact that implementation strategies are often not defined or are described in insufficient detail to allow researchers and other implementation stakeholders to replicate the strategies [6]. Taken together, these deficiencies complicate the transfer of implementation science knowledge from researchers to clinical partners.

Efforts have been made to improve the conceptual clarity of implementation strategies. Taxonomies of implementation strategies *e.g.*, [9,14,15] and behavior change techniques [16] have been developed to encourage more consistent use of terms and definitions in the published literature. Additionally, several groups have advanced reporting guidelines and advocated for the improved reporting of implementation strategies [6,10,17,18]. Despite these important attempts to improve conceptual clarity, there remain several opportunities for improvement. For instance, existing taxonomies of implementation strategies have not been adapted to specific contexts, have not effectively incorporated the voice of practitioners, and have not been developed using rigorous mixed methods. The ERIC process will address these gaps. First, we will apply a published taxonomy of implementation strategies [9] to VA mental health service settings. Second, we will deliberately integrate the perspectives of experts in both implementation science and clinical practice to improve communication between researchers and ‘real world’ implementers and to increase the chances that a full range of strategy options is considered. Finally, we will establish consensus on implementation strategy terms and definitions and develop conceptually distinct categories of implementation strategies. Pursuing these opportunities for improvement will increase the rigor and relevance of implementation research and enable selection of appropriate, feasible and effective implementation strategies to get new EBPPs into routine clinical practice.

### Challenges associated with the selection of implementation strategies

Identifying and selecting implementation strategies for use in research and practice is a complex and challenging process. There are several reasons for this: the limited extent to which the empirical literature can be used to justify the selection of one strategy over another for a given implementation effort; challenges associated with considering dozens of potentially relevant strategies for a particular change initiative; the underutilization of theory in implementation research and practice; challenges associated with the characteristics of different EBPPs; and the wide array and complexity of contextual factors that strongly influence the success or failure of specific implementation strategies.

The evidence base for specific implementation strategies has advanced considerably [19,20]; however, it rarely provides adequate guidance regarding which strategies are likely to be effective in specific circumstances. This is particularly true in mental health and social service settings where the number of randomized controlled trials and head-to-head comparisons of implementation strategies pales in comparison to those conducted in other medical and health service settings [21-25]. In addition to the fact that it is well established that training clinicians to deliver complex psychosocial treatments (*e.g*., via training workshops) is insufficient in isolation [26], evidence is lacking about the types of implementation strategies that are necessary to supplement training at the client, clinician, team, organizational, system, or policy levels. The dearth of economic evaluations in implementation research also makes it difficult to ascertain the costs and benefits of specific implementation strategies [27,28].

The empirical evidence for specific implementation strategies is difficult to summarize because of the large number of strategies listed in the literature and the lack of consistency of their defined features [5]. A recent paper identified 68 discrete implementation strategies [9]. This high number of strategies presents implementation researchers and clinical managers with the challenge of deciding which ones are relevant strategies to meet their particular implementation goals. Market researchers have developed an approach to address these complex types of decisions that involve a wide array of choices using ‘choice menus.’ Choice menus structure options in a way that allow decision-makers to consider a large range of choices in building their own products or solutions. As a result, mass customization of consumer products has expanded greatly over the last decade [29]. Choice menus highlight a trade-off: more choices give decision-makers greater flexibility but simultaneously increase the complexity (*i.e.*, cognitive burden) of making decisions [30]. However, decision-makers with high levels of product expertise consider large choice menus less complex than do consumers with low levels of product expertise [31]. Likewise, choice menus can be used to structure large numbers of implementation strategies, particularly when used by decision-makers with expertise in implementation. Given the level of content expertise implementation scientists and clinical managers bring to quality improvement initiatives, choice menus can be an effective tool for selecting among the dozens of potentially relevant implementation strategies for a particular change initiative.

In the absence of empirical evidence to guide the selection of strategies, one might turn to the considerable number of theories and conceptual models pertaining to implementation in order to guide the selection of strategies [32,33]. However, reviews of the published literature have found that theories and models have been drastically underutilized [23,34,35]. This limits our ability to understand the mechanisms by which implementation strategies exert their effects, and ultimately, how, why, where, when and for whom implementation strategies are effective. The underutilization of theory may also be indicative of limitations of the theories and models themselves [36,37], and signal the need to develop more pragmatic tools that can guide the selection of implementation strategies in practice settings.

The characteristics of the EBPPs themselves present another challenge to the selection of implementation strategies [32,38,39]. Different types of EBPPs often require unique implementation strategies to ensure their implementation and sustainment [40,41].

Finally, contextual variation often has immense implications for the selection of implementation strategies [42]. For instance, settings are likely to vary substantially with regard to patient characteristics [43,44]; provider-level factors such as attitudes toward EBPPs [45]; organizational-level characteristics such as culture and climate [46], implementation climate [47], organizational readiness for change [48], leadership [49,50], capacity for sustainability [51,52], and structural characteristics of the organization [53]; and systems-level characteristics such as policies and funding structures that are facilitative of the EBPP [54]. It is likely that implementation strategies will need to be tailored to address the specific barriers and leverage existing facilitators in different service settings [2,55,56].

Given the complexity of choosing implementation strategies and the absence of empirical data that can guide such a selection, there is a need for, first, methods that can improve the process of selecting implementation strategies; and second, recommendations for the types of strategies that might be effective within specific settings given variation with regard to both context and the EBPPs being introduced. This study will address both needs through the use of an innovative method for selecting implementation strategies, and advancing recommendations for the types of strategies that can be used to implement three different EBPPs within VA mental health service settings.

### Study aims

This mixed methods study will address the aforementioned gaps related to conceptual clarity and selection of implementation strategies through the following aims:

### Aim 1

To establish consensus on a common nomenclature for implementation strategy terms, definitions and categories that can be used to guide implementation research and practice in mental health service settings.

### Aim 2

To develop a set of recommendations that specifies implementation strategies likely to be effective in integrating EBBPs into VA mental health service settings.

## Methods/Design

### Overview

The ERIC process involves a four-stage sequential mixed methods design (qualitative → QUANTITATIVE) [57]. Stages 1 and 2 are used to establish expert consensus on a common nomenclature for implementation science (Aim 1). Stages 3 and 4 build upon the earlier stages and are used to develop expert recommendations regarding how to best match discrete implementation strategies to high priority implementation scenarios in mental health (Aim 2). Table 1 provides an overview of the study’s aims and stages. Qualitative methods are used to develop expert recommendations, and quantitative methods are used to guide the recommendations by obtaining ratings of implementation strategies (alone and as applied to example implementation scenarios), providing structured feedback to the expert panel, and characterizing the consensus process.

**Table 1:** Overview of the four stages of the ERIC process

	**Stage**	**Input**	**Task**	**Output**
**Aim 1**	Stage 1	Refined compilation of discrete implementation strategies	Modified Delphi, 2 feedback rounds and consensus meeting	•Expert consensus on key concepts (definitions & ratings)
	Modified Delphi			
	Stage 2	Post-consensus compilation of discrete implementation strategies	Sort the strategies in to subcategories; rate each strategy in terms of importance and feasibility	•Weighted and unweighted cluster maps
	Concept Mapping			•Ladder maps
				•Go-zone graphs
				•Importance and feasibility ratings for each strategy
**Aim 2**	Stage 3	•Discrete implementation strategies	Essential ratings are obtained for each strategy for three temporal frames given each scenario	For each practice change:
	Menu-Based Choice	•Practice change narrative		•Relative Essentialness Estimates for each strategy given each scenario
		•Narratives of contextual variations of practice change scenarios		•A rank list of the most common strategy recommendation combinations
				•A summary of strategies that may serve as compliments and substitutes for each other
	Stage 4	•Menu-Based Choice data summaries for each scenario	Facilitated discussion; live polling of consensus reached during discussion	For each practice change:
	Facilitated Consensus Meeting	•Importance and feasibility ratings from the concept mapping task		•Expert consensus regarding which discrete implementation strategies are of high importance
				•Context specific recommendations

### Study participants

Purposive sampling will be used to recruit an Expert Panel composed of implementation science experts and VA mental health clinical managers to participate in each of the four stages. The Expert Panel will be recruited using a snowball reputation-based sampling procedure in which an initial list of implementation science experts will be generated by members of the study team. The study team will target members of several different groups based on their substantial expertise in implementation research. These groups include: the editorial board for the journal ‘Implementation Science,’ implementation research coordinators (IRCs) for VA QUERIs [4], and faculty and fellows from the Implementation Research Institute [58]. Nominees will be encouraged to identify peers with implementation science expertise as well as clinical management expertise related to implementing EBBPs [59]. The groups identified to seed the snowball sampling method will be intentionally diverse to ensure adequate recruitment of VA and non-VA implementation experts. This approach to recruit a purposive sample is consistent with the qualitative methods employed in the study design [60].

Recruitment will target 25% to 50% clinical manager representation to ensure that recommendations in Aim 2 reflect the expertise of both scientists and clinical managers. The minimum total enrollment target for the Expert Panel is 20. There are only marginal increases in the reliability of expert consensus methods after sampling crosses the threshold of 12 participants [61], and a minimum enrollment of 20 should ensure adequate saturation in qualitative analyses for the expert consensus and recommendation meetings in Stages 1 and 4 [62]. Implications of this sample size target for Stages 2 and 3 will be discussed as their respective methods are presented. Only individuals residing in the four primary time zones of North America (*i.e.*, Eastern through Pacific) will be recruited to minimize scheduling conflicts for the live webinar portions of the study.

### Stage 1: modified Delphi process

Stage 1 involves a three-round modified Delphi process [63]. The first two rounds involve surveys delivered through an online survey platform. Panelists will have two weeks to complete each of the online surveys. The Powell *et al*. [9] compilation of 68 implementation strategies will be the foundation for the Round 1 survey. Grounding the initial Delphi round in concepts derived from the literature is more efficient for panels composed of experts who are familiar with the key concepts versus using multiple Delphi rounds for the panelists to generate the key concepts on their own [64].

Section 1 of the Round 1 survey will present each implementation strategy accompanied by its definition [9], a synonym response box, and an open comments response box. Panelists will be presented with the following instructions:

The table below lists a number of discrete implementation strategies along with their definitions. For the purposes of this exercise, discrete implementation strategies are defined as single actions or processes that may be used to support implementation of a given evidence-based practice or clinical innovation. The discrete implementation strategies listed below were taken from Powell *et al.*[9].

Before reviewing these terms, take a moment and think of all the implementation projects with which you are most familiar. Taking all of these experiences into consideration, please review the list of discrete implementation strategies below.

If a listed strategy is very similar to other strategies (by a different name) with which you are familiar, please enter the names of the similar strategy(ies) in the “synonyms” text box. If you have any additional thoughts or concerns regarding the definition provided for a given implementation strategy (*e.g.*, specificity, breadth, or deviation from a familiar source), please type those comments into the “Comments” text box.

Section 2 of the Round 1 survey will provide panelists with the opportunity to propose additional strategies that were not included in Powell *et al*. [9]. The instructions for this section are as follows:

Again considering all of your experiences with implementation initiatives, and considering the list of discrete implementation strategies above from Powell, *et al.*[9], can you think of any additional strategies that were not included in the list? If so, please provide the name of the strategy below and provide a definition (with reference citation) for the strategy. If you feel the list of terms in Section 1 was adequately comprehensive, you can leave this section blank.

In Round 2 of the Delphi process, the panelists will be presented with another survey with the implementation strategy terms and definitions from Round 1 as well as a summary of the panelists’ comments and additional strategies. This will include a quantitative characterization where possible (*e.g.*, 72% of panelists made no comment). Several methods will be used to provide participants with greater structure for their responses in Round 2. First, the core definition from Powell *et al.*[9] will be separated from its accompanying ancillary material, allowing for the feedback from the first round to be summarized in terms of concerns with the core definition, alternative definitions, and concerns or addendum to the ancillary materials for the strategy. Second, the strategy terms in Round 2 will be grouped by the types of feedback received in Round 1 (*e.g*., strategies where alternate definitions are proposed, strategies where comments only concerned modifications or addenda to ancillary material). Panelists’ responses in Round 2 will be used to construct a final list of strategies and definitions for the consensus meeting in Round 3. Terms and definitions for which there are neither alternative definitions proposed nor concerns raised regarding the core definition will be considered ‘acceptable’ to the expert panel and will not be included in Round 3 voting. A full description of the instructions provided in Round 2 is provided in Additional file 1.

In Delphi Round 3, members of the study team will lead the Expert Panel in a live polling and consensus process utilizing a web-based interactive discussion platform. Prior to the webinar, panelists will be emailed a voting guide describing the voting process (see Additional file 2) and a ballot that will allow them to prepare their likely responses in advance (see Additional file 3). In Round 3, each implementation strategy term where concerns are raised regarding the core definition will be presented along with alternative definitions proposed from earlier rounds. Terms involving only one alternative definition will be presented first, followed by those with multiple alternatives proposed, and finally, any new terms proposed by the panelists will be presented.

**Figure 1:**
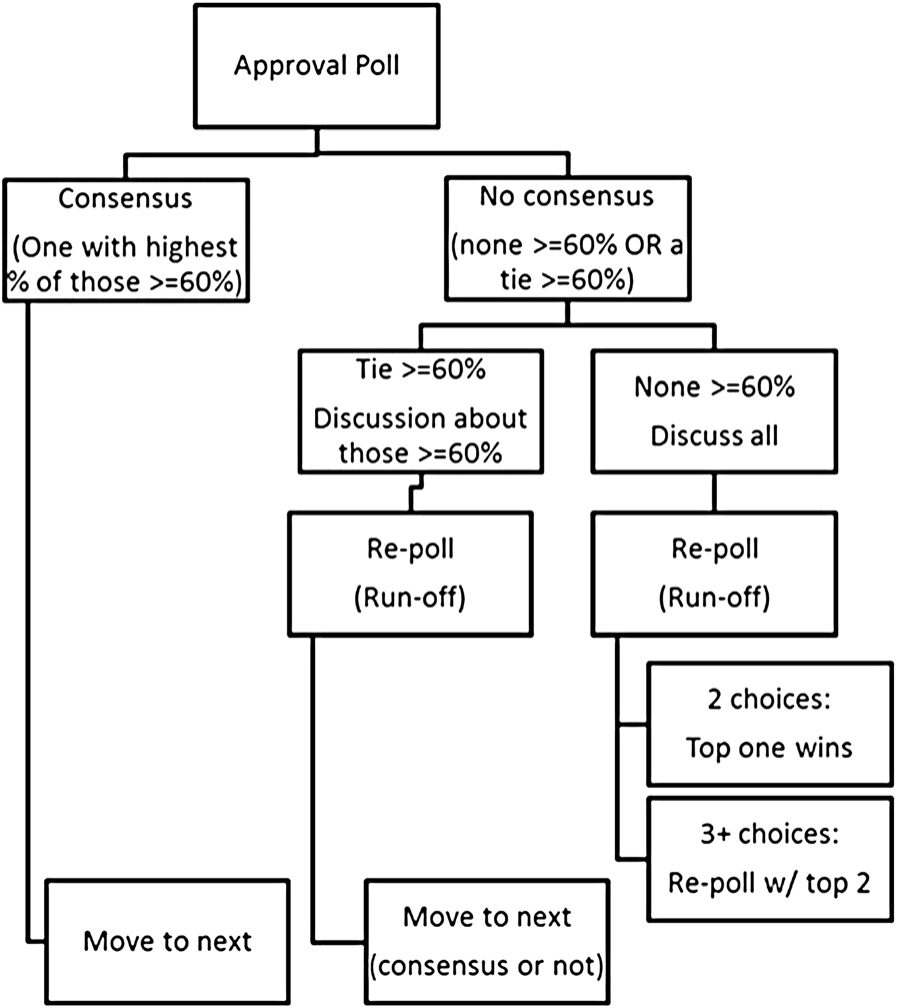
**Overview of the voting process in the final round of the modified Delphi task. ***Note.* In the third and final round of the modified-Delphi task, expert panelists will vote on all strategies where concerns were raised regarding the core definition in the first two online survey rounds. For each strategy, the original and proposed alternate definitions will be presented for an approval poll in which participants can vote to approve all definition alternatives that they find acceptable. In the first round of voting, if one definition receives a supermajority of votes (≥60%) and receives more votes than all others, that definition will be declared the winner and the poll will move to the next term. If there is no consensus, a five-minute discussion period is opened. When the discussion concludes, a run-off poll is conducted to determine the most acceptable definition alternative.

The Voting Guide (Additional file 2) and the webinar introductory materials will provide an overview of the voting process (see Figure 1). The initial vote will be an ‘approval vote,’ where panelists can approve of as many definitions (original and alternative) as they wish. Approval voting is useful for efficiently identifying the most acceptable choice [65], and it also allows for the characterization of approval for the original definitions from Powell *et al.*[9] even when these definitions do not receive the highest rate of approval.

In the first round of voting, if one definition receives a supermajority of votes (≥60%) and receives more votes than all others, that definition will be declared the winner and the poll will move to the next term. Approval poll results will be presented to the panelists in real time. If there is no clear supermajority winner, then panelists will have the opportunity to discuss the definitions. Panelists will indicate whether they would like to talk using a virtual hand raise button in the webinar platform. When addressed by the webinar moderator, the participant will have up to one minute to make comments. Discussion will be limited to five minutes per strategy. This discussion duration was chosen for two reasons. First, Rounds 1 and 2 of the modified Delphi process provide participants with the opportunity for unlimited comments, and this feedback influences what is provided in Round 3. Second, the Round 3 webinar will be targeted to last about 60 minutes to improve panelist participation rate and minimize participant burden.

The second round of voting involves a ‘runoff vote’ in which participants will select only their top choice. If there are only two choice alternatives, then the definition receiving the most votes will be declared the winner. If there are three or more choices, two rounds of runoff voting will occur. The first runoff round will determine the top two definitions for the strategy, and the second runoff round will determine the winner. If a tie occurs between the original and alternative definition in the runoff round, the definition already published in the literature will be retained.

For strategies introduced by the expert panel in modified Delphi Rounds 1 and 2, the approval poll will include a ‘reject’ option for the proposed strategy. A supermajority (≥60%) of participants will be needed to reject a proposed strategy. Aside from the reject option, the same approval and runoff voting procedures will be followed as described above.

### Stage 2: Concept mapping

A practical challenge faced when asking experts to consider a large number of concepts while making recommendations is how to structure the presentation of the concepts to minimize the cognitive burden of an already complex task. One strategy to ease cognitive burden when making recommendations is to place strategies into categories to facilitate the consideration of strategies that are similar. The purpose of Stage 2 is to develop categorical clusters of strategies based on how the expert panelists view the relationships among the strategies.

To achieve this purpose, a concept mapping exercise will be used. Concept mapping is considered a substantially stronger methodological approach for characterizing how complex concepts are organized than less structured group consensus methods [66]. Concept mapping in this project will utilize the Concept Systems Global MAX© web platform for participation and data analysis. Participants will first be asked to sort virtual cards of strategies into piles that make sense to them and provide names for the piles created using the web-based platform [67]. Then, panelists will rate each discrete implementation strategy in terms of its importance and feasibility [68-70]. The instructions for the importance rating will be as follows:

Please select a number from 1 to 5 for each discrete implementation strategy to provide a rating in terms of how important you think it is. Keep in mind that we are looking for relative importance; use all the values in the rating scale to make distinctions. Use the following scale: 1 = Relatively unimportant; 2 = Somewhat important; 3 = Moderately important; 4 = Very important; 5 = Extremely important.

Third, participants will provide a feasibility rating for each strategy. The instructions for the feasibility rating were as follows:

Please select a number from 1 to 5 for each discrete implementation strategy to provide a rating in terms of how feasible you think it is. Keep in mind that we are looking for relative feasibility; use all the values in the rating scale to make distinctions. Use the following scale: 1 = Not at all feasible; 2 = Somewhat feasible; 3 = Moderately feasible; 4 = Very feasible; 5 = Extremely feasible.

Prior to participating, panelists will be provided with an instruction sheet (Additional file 4) and the final compilation of the discrete implementation strategies and their core definitions from Stage 1.

The study’s planned minimum enrollment of 20 is above the recommended sample size for concept mapping (≥15) [71]. In this stage, multidimensional scaling and hierarchical cluster analysis will be used to characterize how implementation terms were clustered by panelists, providing the opportunity to quantitatively characterize the categories of terms developed by the panel in terms of how they were rated on key dimensions.

Final data analyses will include visual summaries of data including weighted and unweighted cluster maps, ladder graphs, and go-zone graphs, all specific tools from the web platform used for this analysis [66,68]. Cluster maps provide a visual representation of the relatedness of concepts, and weighted cluster maps are used to depict how concepts within a cluster were rated on key dimensions (*e.g.*, importance). Ladder graphs provide a visual representation of the relationship between dimensions of a concept (*e.g.*, importance and feasibility, importance and changeability). Go-zone graphs are useful for illustrating the concepts that are most actionable (*e.g.*, high importance and high feasibility) and which concepts are less actionable (low importance and low feasibility). Bridge values (*i.e.*, quantitative characterizations of how closely individual concepts within a cluster are related) will also be reported. These summaries will be provided to the Expert Panel for consideration while participating in Stage 3 activities.

### Stage 3: menu-based choice tasks

Stage 3 involves Menu-Based Choice (MBC) tasks. MBC tasks are useful for providing a context rich structure for making decisions that involve multiple elements. This method emulates naturalistic choice conditions and allows respondents to ‘build their own’ products. To our knowledge, this is the first time an MBC task has been used in an expert recommendation process. We decided to utilize this method because of its transparency, structural characteristics that support decision-making involving a large number of choices, and the ability to quantitatively represent the recommendations. The latter component, described below, will support a more structured dialogue for the final meeting to develop recommendations in Stage 4.

In the MBC tasks, panelists will be presented with the discrete strategies refined in Stages 1 and 2, and they will build multi-strategy implementation approaches for each clinical practice change being implemented. Within each practice change, three scenarios will be presented that vary in terms of implementation relevant features of the organizational context (*e.g.*, organizational culture, leadership, evaluation infrastructure) [44]. Project staff will construct the practice setting narratives using the following multi-stage process. First, a VA Mental Health QUERI advisory committee comprised of operations and clinical managers will be asked to identify high priority and emerging areas of practice change for VA mental health services (*e.g.*, metabolic monitoring for patients taking antipsychotics, measurement-based care, psychotherapy practices). Second, project staff will construct narrative descriptions of specific practice changes (*e.g.*, improving safety for patients taking antipsychotic medications, depression outcome monitoring in primary care mental health, prolonged exposure therapy for treating post-traumatic stress disorder). Third, project staff will construct narrative descriptions of implementation scenarios with varying organizational contexts. Fourth, practice setting narratives will be sent to clinical managers who will be asked to: rate how similar each setting narrative is to their own clinical setting; rate how similar each setting narrative is to other known clinical settings at the VA; and identify descriptors that would improve the narrative’s match with their own or other known clinical settings at the VA. This feedback will be used to refine the content of the MBC tasks before distribution to the expert panel.

**Figure 2:**
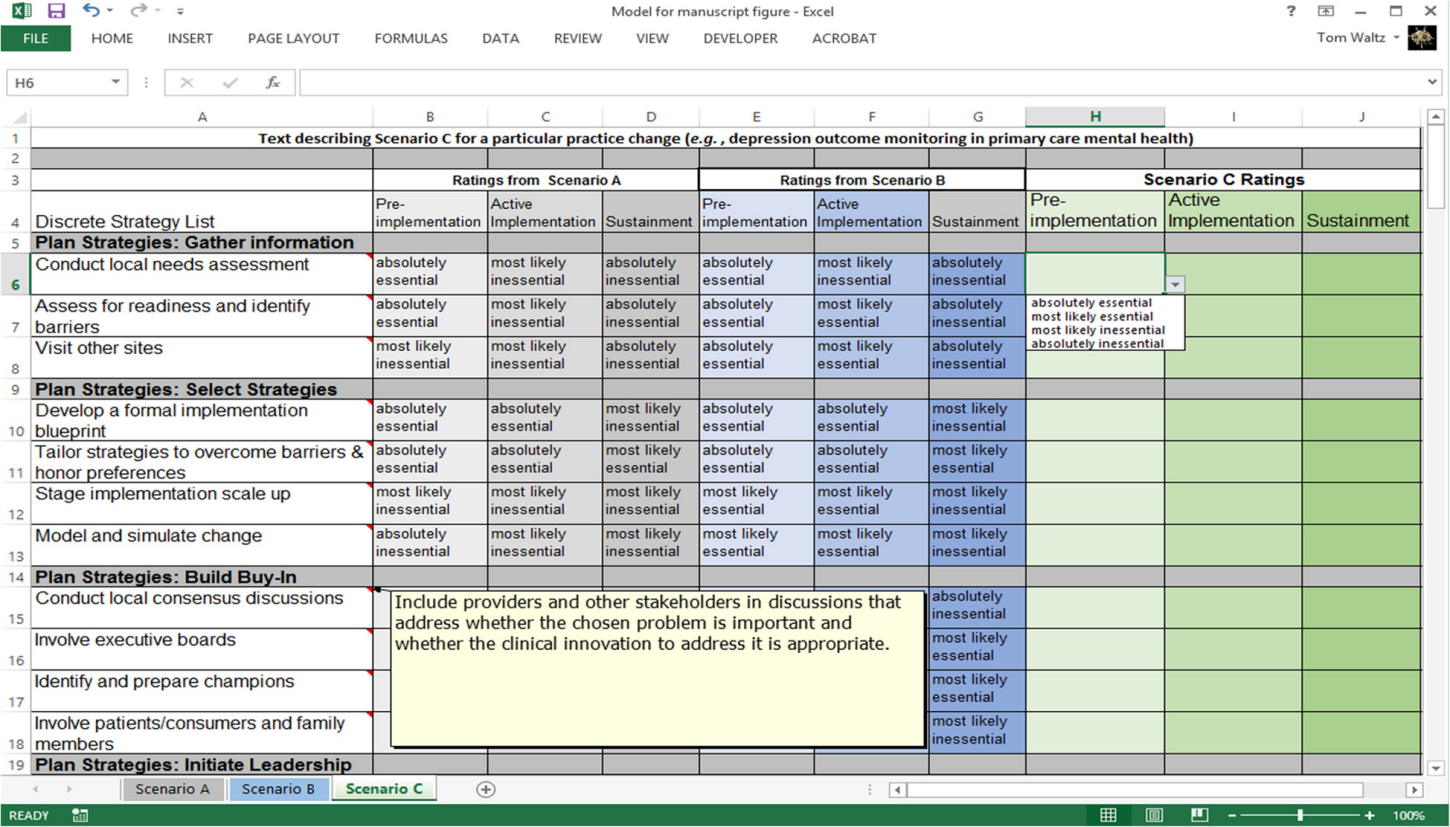
**Screenshot of the MBC task worksheets. ***Note.* Each practice change will have an Excel workbook that has a separate worksheet for each of three scenarios (*i.e.,* Scenario A, Scenario B, Scenario C), with each practice context having different barriers and facilitators. Several features support multifaceted decision-making while completing the task. First, all of the discrete implementation strategies developed in ERIC Stage 1 will be listed in the first column, and sorted into categories based on ERIC Stage 2 Concept Mapping data. Further, for each strategy, a comment box containing the definition for the term appears when the participant moves their cursor over the strategy’s cell. In Figure 2, the ‘Conduct local consensus discussions’ (cell A15) definition box has been made visible. Second, the participant response options are provided in a drop-down menu format to prevent data entry errors. In Figure 2, cell H6 has been selected so the drop-down menu is visible. Third, participants will be encouraged to complete their recommendations for Scenarios A through C sequentially. After the recommendations have been made for Scenario A, these will remain viewable on the worksheet for Scenario B, and the recommendations for Scenarios A and B remain viewable on the Scenario C worksheet, as seen in Figure 2. This supports the participants in efficiently making recommendations considering the current context (Scenario C) while comparing and contrasting these recommendations with those provided for Scenarios A and B, where different combinations of barriers and facilitators are present. Finally, different hues of the response columns are used to visually separate the recommendations for the three contexts with ‘Pre-implementation’ having the lightest shade and ‘Sustainment’ having the darkest.

In the MBC tasks, panelists will indicate how essential each discrete implementation strategy is to successfully implement the practice changes described in each narrative, taking care not to burden the care system with unnecessary implementation tasks. Essential ratings (*i.e.*, absolutely essential, most likely essential, most likely inessential, absolutely inessential) will be dichotomized as essential and inessential for primary analyses used for panelist feedback. Panelists will provide essential ratings separately for three temporal frames (*i.e.*, pre-implementation, implementation, and sustainment) for each scenario. Strategies will be organized into clusters consistent with the categories identified in Stage 2 to help decrease the cognitive burden of this task [72]. This information will be placed in structured spreadsheets that support participants in considering multiple implementation strategies simultaneously. This structure is designed to improve participants’ ability to consider each strategy recommendation in relation to similar strategies while being able to view whether their recommendations are consistent or change based on timing and contextual features of each scenario (see Figure 2).

Within each scenario of each practice change, a Relative Essentialness Estimate (REE) will be calculated for each discrete implementation strategy to characterize participant recommendations. REEs are based on aggregate zero-centered log-count analyses of the recommendation frequency data. This type of analysis provides a nonparametric characterization of the observed frequency of recommendations where a value of 1 represents the highest recommendation rate and 0 represents the lowest recommendation rate for the sample. This type of analysis will be used because it is appropriate for studies with 20 or more participants [73,74]. In Stage 4, REEs for each strategy will be presented to participants accompanied by the corresponding importance and feasibility ratings obtained in Stage 2 (context independent ratings). Count-based analyses will be used to characterize the most commonly selected combinations of essential strategies for each scenario, and graphical and descriptive analyses of these counts will also be presented in Stage 4. The relationship between discrete strategies as compliments or substitutes will be analyzed through dividing the actual joint probabilities of strategies by expected joint probabilities (assuming independence) [73]. Complementarity and substitutability numbers will be used as discussion points in Stage 4.

### Stage 4: Web-based facilitated expert recommendation process

A live web-based facilitated expert recommendation process will be employed in Stage 4. Separate webinars will be hosted for each of the three practice changes. Prior to the webinar, respondents will be provided with the following materials for each scenario: a description of the scenario for continued reference; a personal summary of the essential ratings he or she provided for each implementation strategy at each temporal phase of implementation; and group data describing numerical and graphical descriptive analyses of the most commonly selected combinations of essential strategies, itemization of strategies qualifying as substitutes or compliments, the REE of each strategy, and Stage 2 importance and feasibility ratings of each strategy. During the interactive webinar, study investigators will facilitate a general discussion of the summary material provided to panelists in preparation for developing recommendations for which implementation strategies are essential at each of the three temporal phases in the particular scenarios. This will be followed by scenario-specific facilitated discussions of the top five essential strategy combinations obtained in Stage 3. Live polling will be used to document the degree of consensus for the final recommendations for each scenario. Polling will commence one scenario at a time, addressing each temporal phase of implementation separately, one conceptual cluster of strategies at a time, presenting the top five essential strategy combinations plus any additional combinations identified as highly preferable during the facilitated discussion. Poll results will be used to characterize the expert panel’s rate of consensus for the final set of recommendations regarding which discrete strategies are essential for each phase of implementation for a particular implementation scenario.

### Trial status

The Institutional Review Board at Central Arkansas Veterans Healthcare System has approved all study procedures. Recruitment and data collection for this study began in June of 2013.

## Discussion

This multi-stage mixed methods study will produce consensus on a common nomenclature for implementation strategy terms, definitions, and their categories (Aim 1) and yield contextually sensitive expert recommendations specifying which implementation strategies are likely to be effective in supporting specific practice changes (Aim 2) as listed in Table 1. This study will use innovative technology to engage multiple stakeholder experts (*i.e.*, implementation scientists and clinical managers). First, the three-round modified Delphi procedure will involve input through two rounds of online surveys followed by one virtual webinar meeting, targeting only the strategies where consensus concerns were noted in the first two rounds. The virtual nature of this and subsequent ERIC activities decreases the logistical hurdles involved in obtaining involvement from high-level stakeholders.

Second, a web-based concept mapping platform will be used to capture how expert panelists rate the importance and feasibility of the implementation strategies, as well as how the strategies are conceptually organized. This latter output is particularly important because the number of discrete implementation strategies that can be considered for any particular practice change initiative is vast, and conceptual organization of the strategies is essential for supporting the expert recommendation process.

Third, while the concept mapping exercise includes an assessment of each discrete implementation strategy’s importance and feasibility, these represent global ratings rather than context-specific recommendations. To obtain preliminary, context-specific recommendations for three phases of implementation (pre-implementation, active implementation, and sustainment), a series of MBC tasks will elicit expert recommendations for collections of recommended strategies to address the needs for each of three real-world implementation scenarios. Aggregate data from this exercise will produce quantitative characterizations of high and low levels of consensus for individual strategies at each phase of implementation for each scenario.

Finally, using the data from the MBC task, a webinar-based facilitated discussion will focus on the top suggested strategy combinations followed by voting for recommendations. The structured use of technology in this process allows for experts to participate in the majority of activities on their own time, with only the webinars requiring real-time participation.

While this particular application of the ERIC process focuses on the implementation of EBPPs in mental health service settings within the VA, these methods are suitable for other practice areas. It is worth emphasizing that the ERIC process is essentially two coordinated packages: the first for obtaining consensus on a common nomenclature for implementation strategy terms, definitions and categories; the second for developing context-sensitive expert recommendations from multiple stakeholders. Future studies considering using ERIC may only need to utilize Aim 2 methods (MBC and facilitated webinar) to develop expert recommendations. Regardless of the clinical area or implementation gap being addressed, ERIC-based recommendations fill a gap in the evidence base for designing implementation supports and represent unique opportunities for investigating implementation efforts.

We anticipate that the value of the products produced by this process (*i.e.*, the compendium of implementation strategies, a refined taxonomy of the strategies, and context specific expert recommendations for strategy use, see Table 1) will be of immediate use in VA mental health service settings and provide a template approach for other settings.

## Abbreviations

EBPP: Evidence-based programs and practice; ERIC: Expert recommendations for implementing change; MBC: Menu-Based Choice; QUERI: Quality Enhancement Research Initiative; REE: Relative Essentialness Estimate; VA: U.S. Department of Veterans Affairs.

## Competing interests

The authors declare that they have no competing interests.

## Authors’ contributions

TJW and JEK are Co-Principal Investigators of the funded project. JLS, MMM, MJC, and LJD are Co-Investigators. EKP and BJP are consultants. TJW and BJP drafted this manuscript. All authors reviewed, gave feedback, and approved the final version of this manuscript.

## Supplementary Material

Additional file 1 Welcome to ERIC modified Delphi Round 2. Click here for file

Additional file 2 ERIC Voting Guide. Click here for file

Additional file 3 ERIC Voting Notes. Click here for file

Additional file 4 Concept Mapping Instructions for Expert Recommendations for Implementing Change (ERIC). Click here for file
