# Supplementary material for: Expert recommendations for implementing change (ERIC): protocol for a mixed methods study
Source: Implement Sci. 2014 Mar 26;9:39. doi: 10.1186/1748-5908-9-39 (PMC3987065; doi:10.1186/1748-5908-9-39)
Supplement: Additional file 1 — Welcome to ERIC modified Delphi Round 2. [file 1748-5908-9-39-S1.docx]

Additional File 1

**Welcome to ERIC modified Delphi Round 2.**

The ERIC investigative team would like to thank everyone who participated in Round 1. An incredible 77% of invitees responded to the invitation and made contributions.

In Round 1, the text describing the core definition and ancillary material for each strategy were presented as continuous text. In Round 2, the ancillary material is presented separate from the core definition. This is intended to ease the burden of the review and commentary process. The core definition will be fixed by the end of the modified Delphi activities. Ancillary material is intended to be more fluid and accommodate additional guidance and examples regarding enacting the strategy. We expect to aggregate additional ancillary material across the ERIC activities and after the formal activities are completed.

Note that ERIC Part 2 activities (i.e., Menu-Based Choice and consensus webinar) will provide further opportunities for respondents to contribute ancillary material specific to the three practice changes included in those activities. Thus, one strategy for expediting your participation in Round 2 is to reserve ancillary material additions for the Part 2 activities. However, ancillary material comments are still welcome in Round 2.

**In Round 2, the strategies are organized by section based on the types of feedback received in Round 1. An overview of the sections is provided below.**

- **Section 1.** **Terms targeted for voting**. This section contains strategies where respondents provided alternate definitions or other content that will likely be involved in the voting conducted in the consensus webinar. Many of these terms also received ancillary material contributions.
- **Section 2.** **Terms where the core definition raised concerns.** This section contains strategies where respondents noted possible difficulties with the core definition but no alternate definitions were proposed. Many of these terms also received ancillary material contributions.
- **Section 3**. **Terms with no comments about the core definition or ancillary materials.** This section contains strategies where none of the comments suggested changes to the core definition or ancillary material.
- **Section 4.** **Terms with few comments on ancillary materials.**  This section contains strategies where two or fewer respondents suggested ancillary material changes. No changes to the core definition were suggested.
- **Section 5.** **Terms with many comments on ancillary materials.** This section contains strategies where three or more respondents suggested ancillary material changes. No changes to the core definition were suggested.
- **Section 6.** The study information sheet for the ERIC project is provided. This is the same information sheet you received when you were invited to participate in the study. The “Done” button is at the bottom of this section.

**Notes on how Round 1 feedback regarding the strategy descriptions was integrated:**

Comments were triaged as concerning either a) the core definition b) ancillary material, or c) other.

- **Core definition comments**:
  - Comments identifying substantive changes to the definition resulted in proposed alternate definitions, labeled as ALT 1, ALT 2, etc. Terms with these types of suggestions are listed in Section 2.
  - Comments noting concerns with the definition (e.g., clarity, wordsmithing) are listed in Section 3.
  - Note, suggestions regarding merging terms with similar content were noted but no actual merging will occur as part of the modified Delphi process. The Concept Mapping activity will provide an empirical basis for collapsing strategies later in this project.
- **Ancillary material comments** were integrated into the ancillary material narrative.
- **Other comments** contributed to the total comment count provided in the summary data for each strategy. The content of these comments are not included in the summary.
  - Some of these comments were editorial.
  - Incorporation of comments regarding the evidence base or feasibility of the strategies is beyond the scope of this project.
  - Comments that would require the investigators to research the specific details of implementation frameworks were not followed up upon due to time restraints. ERIC investigative team members are interested in relating the strategies included in this project to existing implementation frameworks and these relationships will be explored at another date. In many cases, the source paper for the strategies presented in ERIC (Powell et al., 2012) had referenced the frameworks/approaches suggested (e.g., EPOC). If you feel that an important discrete implementation strategy had been obscured through the compilation process in Powell et al. (2012) please clearly specify the strategy and its definition in your comments in Round 2. The last comment box in Section 2 is for these proposals.

**Synonym data** has been retained for future data analysis. Only in instances where the label for a strategy directly conflicted with the existing literature were alternate labels proposed (in Section 2). Terms where the relationship between the label and the definition were of concern are included in Section 3.

**[Section 1 Materials]**

**Terms targeted for voting**

Comments to these terms involved suggestions for alternative definitions and there was one instance of a strategy label being too specific and a more general label is desired.

This is your opportunity to make further comments or alternate definition suggestions regarding these strategies prior to the consensus webinar where voting will occur.

If you feel an alternative definition for the strategy is warranted, propose one in the respective comment box or endorse one of the alternate (ALT) definitions that are listed below the strategy's Round 1 summary by using the comment box.

If you feel the original definition for the strategy is adequate, you can leave the respective comment box empty.

Ancillary material contributions are welcome but not necessary.

****Reminder. Many Round 1 comments noted the similarity or overlap of many of the strategies. Concept Mapping data from a later phase in this study will be used to provide a quantitative basis for considering the merging of terms.

[the term, its core definition, summary of Round 1 feedback, and a comment box follow each strategy]

**[Section 2 Materials]**

**Terms where the core definition raised concerns**

These are terms where some respondents indicated difficulties with the core definition such as it being too vague. The comments were not specific enough to propose an alternate definition for the strategy.

This is your opportunity to make further comments or alternate definition suggestions regarding these strategies prior to the consensus webinar where voting will occur.

If you feel an alternative definition for the strategy is warranted, propose one in the respective comment box.

If you feel the original definition for the strategy is adequate, you can leave the respective comment box empty.

Ancillary material contributions are welcome but not necessary.

****Reminder. Many Round 1 comments noted the similarity or overlap of many of the strategies. Concept Mapping data from a later phase in this study will be used to provide a quantitative basis for considering the merging of terms.

[the term, its core definition, summary of Round 1 feedback, and a comment box follow each strategy]

**[Section 3 Materials]**

**Terms with no comments about the core definition or ancillary materials**

If respondents commented on these strategies in Round 1, the comments were not clearly about changing the core definition or the addition of ancillary material.

This is your opportunity to make comments or alternate definition suggestions regarding these strategies prior to the consensus webinar where voting will occur.

If you feel an alternative definition for the strategy is warranted, propose one in the respective comment box.

If you feel the original definition for the strategy is adequate, you can leave the respective comment box empty.

Ancillary material contributions are welcome but not necessary. Part 2 of the study will provide opportunities to contribute ancillary material for the strategies in the context of particular implementation initiatives.

****Reminder. Many Round 1 comments noted the similarity or overlap of many of the strategies. Concept Mapping data from a later phase in this study will be used to provide a quantitative basis for considering the merging of terms.

[the term, its core definition, summary of Round 1 feedback, and a comment box follow each strategy]

**[Section 4 Materials]**

**Terms with few comments on ancillary materials**

Respondents indicated no changes to the core definitions of these strategies. A few respondents (<3) did contribute ancillary materials.

This is your opportunity to make further comments or alternate definition suggestions regarding these strategies prior to the consensus webinar where voting will occur.

If you feel an alternative definition for the strategy is warranted, propose one in the respective comment box.

If you feel the original definition for the strategy is adequate, you can leave the respective comment box empty.

Ancillary material contributions are welcome but not necessary. Part 2 of the study will provide opportunities to contribute ancillary material for the strategies in the context of particular implementation initiatives.

****Reminder. Many Round 1 comments noted the similarity or overlap of many of the strategies. Concept Mapping data from a later phase in this study will be used to provide a quantitative basis for considering the merging of terms.

[the term, its core definition, summary of Round 1 feedback, and a comment box follow each strategy]

**[Section 5 Materials]**

**Terms with many comments on ancillary materials**

Respondents indicated no changes to the core definitions of these strategies. Three or more respondents did contribute ancillary materials.

This is your opportunity to make further comments or alternate definition suggestions regarding these strategies prior to the consensus webinar where voting will occur.

If you feel an alternative definition for the strategy is warranted, propose one in the respective comment box.

If you feel the original definition for the strategy is adequate, you can leave the respective comment box empty.

Ancillary material contributions are welcome but not necessary. Part 2 of the study will provide opportunities to contribute ancillary material for the strategies in the context of particular implementation initiatives.

****Reminder. Many Round 1 comments noted the similarity or overlap of many of the strategies. Concept Mapping data from a later phase in this study will be used to provide a quantitative basis for considering the merging of terms.

[the term, its core definition, summary of Round 1 feedback, and a comment box follow each strategy]
