# Supplementary material for: Expert recommendations for implementing change (ERIC): protocol for a mixed methods study
Source: Implement Sci. 2014 Mar 26;9:39. doi: 10.1186/1748-5908-9-39 (PMC3987065; doi:10.1186/1748-5908-9-39)

Additional File 4

**Concept Mapping Instructions for**

**Expert Recommendations for Implementing Change (ERIC)**

1. Log on into the website (<http://www.conceptsystemsglobal.com/ERIC/sort>) using your email address as your user name and “ERIC” as your password.

Note: if you have been a participant in a conceptsystemsgloabl.com based project in the past, your email address may be associated with the password for the previous project. Try “ERIC” first and if you receive an error message, use your previous password to log in.

1. Once you log in to the site, you will need to click on the blue link labeled “Expert Recommendations for Implementing Change (ERIC)” under “My Projects.”
2. The next screen you will see is the informed consent (study information sheet), which you will need to accept in order to enter the project.
3. Upon accepting the project you will see a screen with four sections listed under “Next Steps”:

(1) Participant Questions,

(2) Sorting,

(3) Rating Importance, and

(4) Rating Feasibility.

Note that you can begin on any “step” that you choose, and you need not complete all of the steps in one sitting. You may also save your progress within the sorting and rating steps if you wish to return to finish them at a later time. You can return to your participant account using the link you received in the email and logging in again.

1. “Participant Questions”:

Participant questions include 3 dichotomous (yes/no) questions pertaining to your experience and expertise related to implementation and your affiliation with the VA. Once you have answered each of these questions, the “participant questions” link will be removed from the list of “Next Steps.”

1. “Sorting”:

Note: Before you begin the sorting task, we ask that you review the strategies and definitions file. The sorting task only provides you with the labels for the strategies. You will likely be referencing the definition file often as you engage with the sorting task.

Once you click on the sorting task, there will be a window that provides instructions. We suggest that you read this carefully. In brief, your task is to sort each of the 73 implementation strategies into piles that make sense to you conceptually. You should not create piles according to priority or value, such as “important” or “hard to do.” Also, you should not create “miscellaneous” or “other” piles; rather, you may place strategies in their own piles (i.e., piles where n = 1) if it is unrelated to other strategies. The typical number of plies for projects with similar numbers of statements as ERIC is 5 to 20. You will also have the opportunity to give each pile a name that represents the strategies that you have included. After you have closed the instructions window, you may view the instructions again through the link on the left side of the menu bar.

You may begin sorting the statements in one of two ways. First, you can simply drag one of the strategies listed on the left hand side of your screen over to the “tabletop,” which will automatically create a new pile and prompt you to name that pile. At that time, you can either name the pile or simply click “ok” to approve the default name (“Unnamed Pile 1,” etc.). The other option you have to create a new pile is simply clicking on the “create a pile” button in the menu bar. Once you have a pile started, you can drag additional strategies into that category as you see fit. There are a number of options on the menu bar that may make this task easier (as seen in the screenshot below). In addition to the “instructions” and “create a pile” buttons, you will be able to save regularly by clicking the third button from the left. The “arrange all” button will display your piles in a uniform fashion as seen below. The “minimize all” or “maximize all” buttons may be helpful if you want to clear your tabletop or ensure that all of your piles are visible. You may always edit pile names using the edit pile name button in the menu bar.

Please name your piles. The pile names are included in the data analysis and will serve as the initial suggested labels for the clusters identified in the initial concept mapping analysis.

Finally, the last button will allow you to switch to a dropdown view rather than the tabletop view that serves as the default. NOTE: The system will prompt you to save your work periodically; however, we recommend that you save frequently to avoid losing any of your work. We also recommend that you save before logging out, as it does not seem to do so automatically.


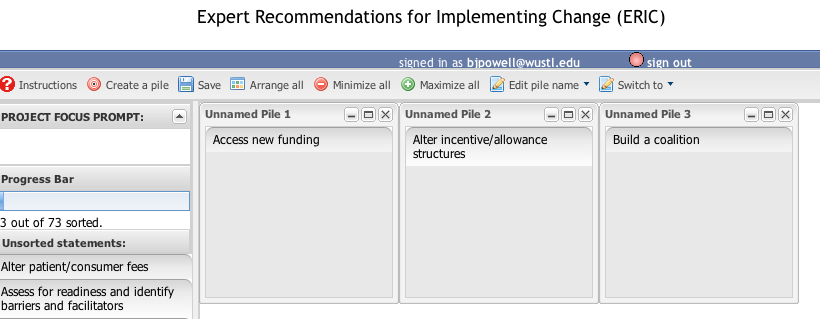


Once you have sorted each of the strategies into a pile, you will see a message on the left hand side of your screen that read, “Congratulations, you have sorted all the statements!” You will then need to click the “save and finish” button at the bottom of that message. Doing so will take you to a page that thanks you for completing the sorting activity, and gives you a link to return to the main page so that you can complete any remaining steps.


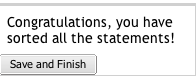


1. “Rating: Importance” and “Rating: Feasibility”:

These two rating sections will take you to a screen that will allow you to rate each strategy on a scale from 1 to 5 according to how important and feasible you think it is. You will enter the actual number for each strategy, and the quickest way to complete this task is to enter a number and then hit your tab key to advance to the next strategy. If you wish to save your ratings in order to return to finish them at another time, you may do so by clicking the “save rating information” button at the bottom of the page. Once you have rated each strategy according to its importance or feasibility and clicked on the “save rating information” button at the bottom of the screen, your answers will be recorded and submitted.

1. Once you have completed all tasks, your screen should look like the one below, with a message thanking you for completing all tasks currently assigned to you. We sincerely thank you for your time and dedication to this project. Please don’t hesitate to let us know if you have any trouble with the Concept Systems Global MAX© software.


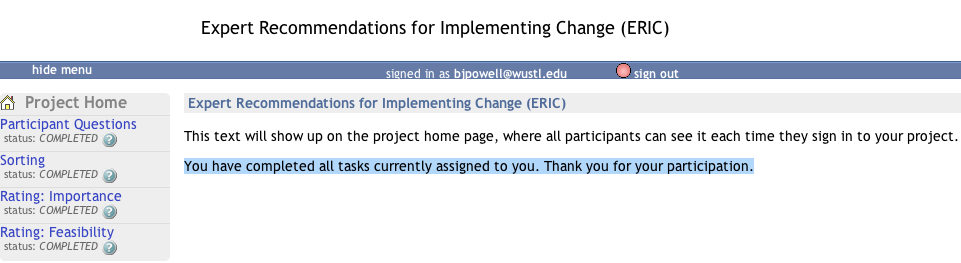

Supplement: Additional file 4 — Concept Mapping Instructions for Expert Recommendations for Implementing Change (ERIC). [file 1748-5908-9-39-S4.docx]
